# Supplementary material for: Modeling of Human Rabies Cases in Brazil in Different Future Global Warming Scenarios
Source: Int J Environ Res Public Health. 2024 Feb 11;21(2):212. doi: 10.3390/ijerph21020212 (PMC10888213; doi:10.3390/ijerph21020212)
Supplement: Supplementary file 1 [file ijerph-21-00212-s001.zip › ijerph-2821255-supplementary.pdf]

Modeling of human rabies cases in Brazil in different future global warming scenarios

Supplementary materials

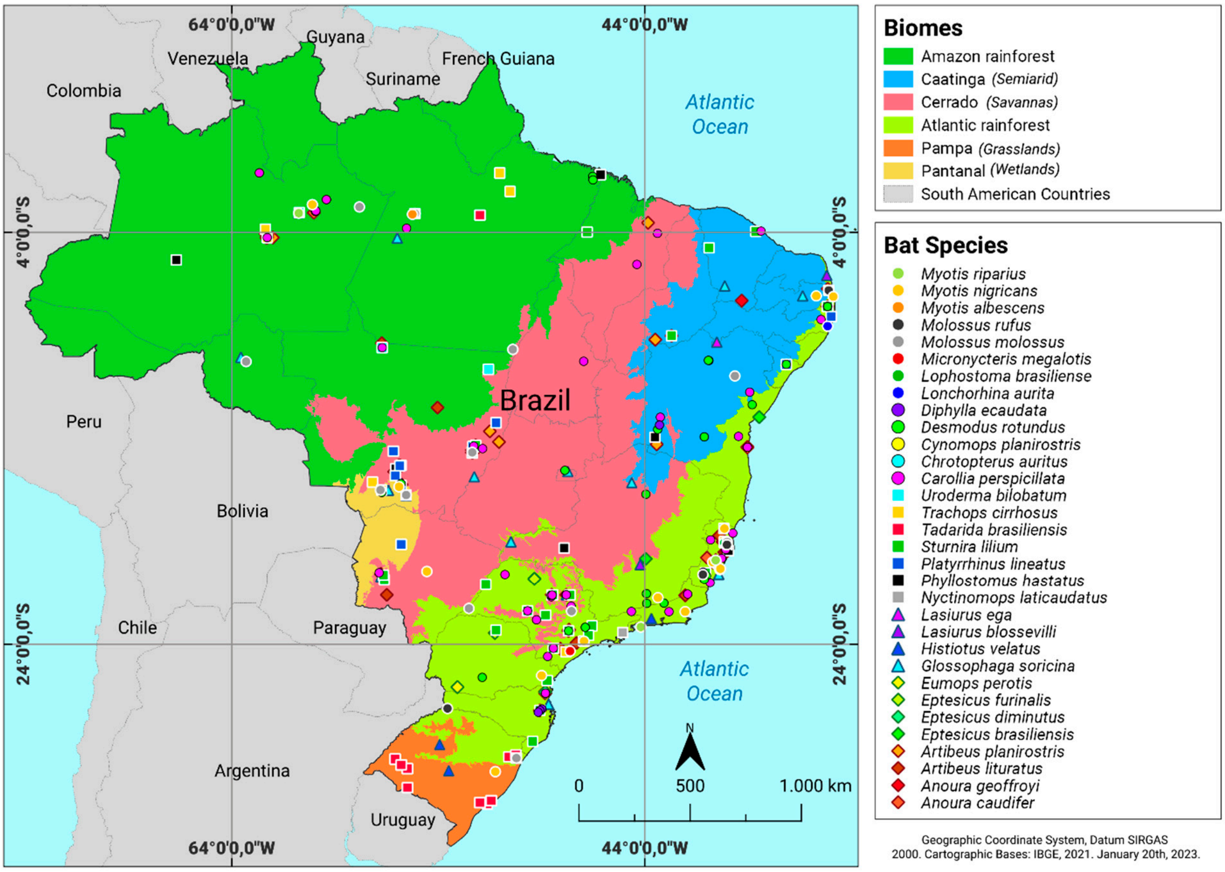

Figure S1. Brazilian biomes and respectives bat species distribuition that tested positive for rabies.
